# The Influence of Alcohol upon the Bodily Defenses against Disease*From the *Journal of Inebriety*.

**Published:** 1907-12

**Authors:** David Paulson

**Affiliations:** Superintendent Hinsdale (Ill.) Sanitarium


### The Influence of Alcohol Upon the Bodily Defenses Against Disease.*

BY DAVID PAULSON, M. D., SUPERINTENDENT HINSDALE (ILL.) SANITARIUM.

One by one solid, substantial, scientific facts are being forged into links in the long chain of convincing evidence that the sum total effect of alcohol is far more disastrous to the human body than it is beneficial.

Years ago it was shown by actual tests measured by a dynamometer that the use of alcohol, instead of increasing muscular strength as had been supposed, actually lessened it.

Then came Kraepelin's incontestable experiments demonstrating that as small a quantity as one-third of an ounce of alcohol produced depressing physical effects which were capable of demonstration by instruments of precision. These results, obtained from a careful study of the influence of alcohol on the muscles, brain and nerves naturally suggested similar effects upon nutrition and also upon the various protective defenses of the body against disease.

Years ago, Boix, after conducting an extensive series of observations, concluded that the sclerotic changes that were formerly attributed to alcohol directly were in reality largely due to the absorption of an increased amount of various toxins resulting from the increased gastro-intestinal disturbances induced by the use of alcohol.

Reid Hunt has recently reported that he found when alcohol was given to rabbits the ethereal sulphates instead of being 1 to 2 per cent were increased to 30 or 35 per cent of the total sulphates.

Accumulating evidence compels us to believe that many of the common chronic diseases, including pernicious anemia and possibly to a certain extent even cancer, are markedly influenced by, if not to a large extent actually due to the constant absorption of excessive quantities of gastro-intestinal toxins.

As at present as never before the searchlight of modern science is being focused upon the tissues themselves, there is revealed to us the tremendous importance of nutritional processes. As Starling expresses it, digestion only furnishes the nutritive bullion from which intracellular digestion must make the real currency for the use of the tissues.

This intracellular digestion is closely identified with those bodily

*From the *Journal of Inebriety*.

defenses against toxins and bacteria. In view of this, some recent investigations made by Reid Hunt of the Public Health and Marine Hospital Service of the United States become of intense interest.

These experiments revealed modifications in the bodily physiological processes from repeated doses of alcohol given in too small quantities to produce the least indications of intoxication. Over considerable periods of time alcohol in gradually increasing amounts was added to oats fed to mice, then the amount of acetonitrile necessary to kill these mice was ascertained, and the interesting observation was constantly noted that it only required about one-half the amount of acetonitrile to kill the mice which had received these moderate quantities of alcohol with their food as was necessary to kill the mice which had been fed on oats free from alcohol. When little larger amounts of alcohol were given to the mice it only required one-third the dose of the drug necessary to kill as compared to those which had not received alcohol. That this effect was not due to any lack of nutrition was made evident from an experiment in which mice were only fed a limited amount of food for a certain period of time, and when the nitrile was injected they recovered from four times the dose which caused death in the alcohol-fed mice.

Guinea pigs were subjected to the same experiment and it was likewise found that alcohol in so small quantities as to produce no noticeable physical effects whatsoever, nevertheless increased their sensibility to acetonitrile just as it did in the mice.

Many have been led to believe that small quantities of alcohol were regarded by the body as a food in just the same sense as sugar and fats, but Reid Hunt found that when mice were fed oats soaked in a solution of dextrose, instead of showing the decrease of resistance to acetonitrile that was noted in the alcohol-fed mice they were actually able to tolerate increased quantities of the drug.

These experiments are too recent to justify us in drawing definite conclusions, but they certainly at least suggest, in the words of the experimenter:

"It is believed that these experiments afford clear experimental evidence for the view that extremely moderate amounts of alcohol may cause distinct changes in certain physiological functions and that these changes may, under certain circumstances, be injurious to the body. The results also afford further evidence that in some respects the action of alcohol as a food is different from that of carbohydrates, and finally that in all probability certain physiological processes in 'moderate drinkers' are distinctly different from those in abstainers."

It would seem that no experiment could demonstrate more conclusively the demoralizing influence of alcohol upon the defensive agents of the body in acute infectious diseases than the investigations made by George Rubin, Pathologist, Rush Medical College, and reported in the *Journal of Infectious Diseases,* May 30, 1904. He studied the influence of alcohol ether and chloroform upon animals who had been subjected to various experimental infections.

Into a rabbit having a leucocyte count of 8600 was injected hyperdermically 1 c. c. of fresh streptococcus culture; the next day the leucocyte count was 15,400; the following day 23,300. This rabbit made a good recovery.

At the same time another rabbit of practically similar weight, having a leucocyte count of 8800, was given 4 c. c. 95 per cent alcohol and then 1 c. c. of streptococcus culture; the next day the leucocyte count, instead of increasing, had fallen to 7000, and the following day it was 7900. Ten days after inoculation this rabbit died in a cachectic state.

Another rabbit with a leucocyte count of 10,500 was given 1 1-3 c. c. pneumococcus culture; the next day its leucocyte count had risen to 11,400; the following day it increased to 18,000. This rabbit recovered without any serious trouble.

At the same time there was injected into another rabbit, whose leucocyte count was 14,000, 3 c. c. of alcohol and 1 c. c. of pneumococcus culture. This rabbit lived only twenty-two hours and immediately after death its leucocyte count had dropped to 1100.

I have only selected a few of the many cases of this interesting set of experiments which Rubin reports, but in every case the narcotized animals died, and in those cases where the control animal died they always survived their fellows a certain length of time and gave evidence of resistance to infection in a higher leucocyte count and by other physical signs.

Rubin obtained the same result when chloroform or ether was used. These experiments admirably confirm the belief that has been entertained by a rapidly increasing number of the leading men in the profession, that alcohol instead of assisting the patient who was suffering with some serious infectious disease really served to destroy his only chance for recovery. And one can not but cherish the unpleasant suspicions that thousands of pneumonia patients to whom have been administered liberal doses of alcohol have as a consequence joined the silent majority as the poor rabbits did, because they did not have sufficient resistance to deliver them from not only the infection but also the toxic effects of the alcohol.

After Rubin had noted the depressing effects of alcohol of leucocytosis, he occasionally observed some cases where the animals died with a fairly high leucocyte count, and the idea suggested itself to his mind that perhaps the alcohol also lessened the ability of the leucocytes to pick up the germs. So carmine was injected into the peritoneal cavity of a rabbit, and twenty-four hours later upon examination of some peritoneal exudate it was found that 14 per cent of the leucocytes contained carmine particles, some of them having as many as eight or ten particles. At the same time a similar rabbit was narcotized with alcohol and a similar amount of carmine was injected. Twenty-four hours later only 6 per cent of the leucocytes had taken up any carmine particles.

These results led Rubin to investigate the number of leucocytes in a series of sixty confirmed drunkards, and he found it to average 5300 instead of the normal of 7500. He also found that the periodical drunkards in this list had 1400 more than the steady drunkards.

This interesting investigation suggests that alcohol not only cripples the leucocytes but also the leucocyte-producing organs.

An editorial in the *Journal of the American Medical Association,* in commenting upon these experiments, states:

"With many physicians alcohol has always been the standby in septic conditions. Patients with septicemia are filled with whisky, despite the depressant effect of large doses of alcohol, as if the alcohol was expected to either kill the bacteria in the circulation or at least to neutralize their toxins. * * * No one will question the greater mortality of pneumonia in alcoholics as compared with that in normal individuals, and pus infections usually procure with excessive virulence in patients with delirium tremens; in general, chronic alcoholism to lower decidedly resistance to infectious diseases. It is, therefore, quite reasonable to question the idea that acute alcoholic intoxication will protect against these same infections."

Snel has shown that alcohol will even suspend immunity that has developed against a specific microbe, and Metchnikoff mentions an investigator who succeeded in killing by cholera infection guinea pigs who had been highly immunized against this disease by merely giving before the inoculation a preliminary injection of small quantities of tincture of opium. This retarded the phagocytic action sufficiently to make the animals lose their immunity and render them an easy prey to the vibrionic infection.

Metchnikoff offers the following comments upon the influence of alcohol on immunity against infection:

"Although the phagocytes belong to thee most resistant elements of our body, yet it is not safe to count on their insensibility towards poisons. We have seen how they are harmed even by small doses of opium. * * * It is well known that persons who indulge too freely in alcohol show far less resistance to infectious diseases, especially to croupous pneumonia, than abstemious individuals. The vaccinations against the hydrophobia carried out on persons bitten by mad animals are almost always successful; but those cases in which the treatment does not stop the outbreak of the disease are most frequently observed in individuals addicted to alcoholism.

"In pursuance of this observation, Delearde, of the Pasteur Institute in Lille, has undertaken a series of experiments which have proved to him that the absorption of alcohol is without a doubt a grave obstacle to the immunization against hydrophobia. At the same time he found that rabbits to which he administered alcohol in the course of immunization against anthrax died of this disease, whilst the control animals, which were given no alcohol, could be vaccinated without any difficulty.

"Abbott has confirmed these experiments by proving that animals, if subjected to the influence of alcohol, become more sensitive to the harmful effects of several microbes, such as streptococci, staphylococci and bacterium coli. Later on, Laitinen carried out a great number of experiments from the same point of view and with similar results. Our interest centers mainly in his experiments on the vaccination against anthrax. To a number of rabbits alcohol was administered for several days in succession; they were then injected subcutaneously with a small dose of the first vaccine of anthrax. Six animals thus treated died after a more or less prolonged illness; all of these contained anthrax bacilli in their blood and organs. Of four control rabbits which received the same dose of the same vaccine, but to which no alcohol had been administered, only one died, whilst the other three enjoyed perfect health. Several other experiments furnished similar results.

"Alcohol, therefore, suppresses the natural immunity of rabbits towards the first vaccine of anthrax. This impairment of their resistance was manifested by the inactivity of their white blood cells; thus the bacilli were permitted to multiply without being checked by a sufficiently strong phagocytic reaction. * * *

"Besides its deleterious influence on the nervous system and other important parts of our body, alcohol, therefore, has a harmful action on the phagocytes, the agents of natural defense against infective microbes. * * *

"As a logical consequence of the experiments on the weakening of immunity under the influence of alcohol, it has been suggested to eschew this substance in the treatment of infectious diseases. Without wishing to enter into a discussion of this question * * * we must strongly insist on the danger of alcoholism with regard to the risistance against pathogenic microbes."

In view of these unquestionable facts which have been developed by the most painstaking experiments, is it not self-evident that the physician who depends upon alcohol in the sick room leans upon a broken reed? It is clear that the body regards alcohol not as an aid in its struggle for existence but as a dangerous foe. As the body is compelled to fight this enemy, its opsonic index may be temporarily increased, and there may be other similar indications of the struggle thus provoked, just as there is when it is compelled to wage a warfare against any other toxins. But such evidence will never lead the observing student of nature and her operations to blindly conclude that they represent anything other than a definite resistance to a toxic agent.

